# Use of Balloon Tamponade Device for Aortoesophageal Fistula: A Case Report

**DOI:** 10.5811/cpcem.62142

**Published:** 2026-08-05

**Authors:** Erich Burton, Trenton Wray

**Affiliations:** University of New Mexico, Department of Emergency Medicine, Center for Adult Critical Care, Albuquerque, New Mexico

**Keywords:** aortoesophageal fistula, esophageal-gastric balloon tamponade, hematemesis, Minnesota tube, thoracic endovascular aortic repair

## Abstract

**Introduction:**

Aortoesophageal fistula is a rare but life-threatening hemorrhagic complication of thoracic endovascular aortic repair. Without intervention, mortality approaches 100%. Our case highlights the utility of using an esophageal-gastric balloon tamponade device for hemostasis.

**Case Report:**

We report the case of a 70-year-old man who presented after a sentinel episode of hematemesis six days after thoracic endovascular aortic repair. Shortly after admission, the patient developed massive hematemesis and hemorrhagic shock from an aortoesophageal fistula. Hemorrhage was temporarily controlled via bedside placement of a Minnesota tube, a type of balloon tamponade device, by two emergency medicine and critical care physicians, allowing for hemostasis, resuscitation, and definitive diagnosis with esophagogastroduodenoscopy. The device successfully bridged the patient to the operating room, where he underwent definitive endovascular control of the hemorrhage and repair of the aortoesophageal fistula.

**Conclusion:**

Our case highlights the utility and pragmatism of using a balloon tamponade device for massive hematemesis in nonvariceal hemorrhage. It also supports the placement of the device for non-variceal hemorrhage by nonspecialists, including critical care and emergency physicians.

## INTRODUCTION

Aortoesophageal fistula is a rare but life-threatening complication of thoracic endovascular aortic repair, with a reported incidence of 1.7–1.9%.^1^,^2^ While the vast majority of hematemesis is related to upper gastrointestinal (GI) bleeds from peptic ulcer disease and esophageal varices, vascular etiologies must also be considered.^3^ This is especially true in patients with a history of thoracic aortic or esophageal interventions. Clinical presentation is variable, ranging from Chiari triad (dysphagia or mid-thoracic back pain, sentinel or herald episode of hematemesis, followed by fatal exsanguination) to fatal hemorrhage.^4^ Without intervention, aortoesophageal fistula with hematemesis is almost uniformly fatal. Therefore, rapid identification and intervention are required. Management becomes difficult when deterioration occurs before the patient has received definitive intervention. This manifests as unmanageable rapid hemorrhage leading to death within minutes.

Temporary hemostatis is vital to bridge patients to definitive therapy. Since decompensation may occur in a variety of clinical environments—ranging from the emergency department (ED) to the intensive care unit (ICU) and operating room— a simple, efficient method of hemostasis that can be readily deployed by nonspecialist clinicians is needed. Off-label use of a Minnesota tube, or similar esophageal-gastric balloon tamponade device, can successfully achieve temporary hemostasis in patients with aortoesophageal fistulas. We report the use of a Minnesota tube for hemostasis in a patient with an aortoesophageal fistula performed by two dual-boarded emergency and critical care physicians, in contrast to other case reports in which the procedure was performed by surgeons or gastroenterologists.^5^–^7^ Our case supports that placement of a balloon tamponade device for aortoesophageal fistula by nonsubspecialists can be done quickly, effectively, and safely. It also supports the feasibility of placing this device at bedside, without endoscopy, extending applicability to emergency and critical care environments. This contrasts with other case reports where endoscopy was used to assist placement.^5^–^7^ Further, because hemostatis is typically unachievable via esophagogastroduodenoscopy (EGD), our case is consistent with previously reported cases of using a balloon tamponade device as a bridge to definitive surgical intervention.

## CASE REPORT

**Image 1:**
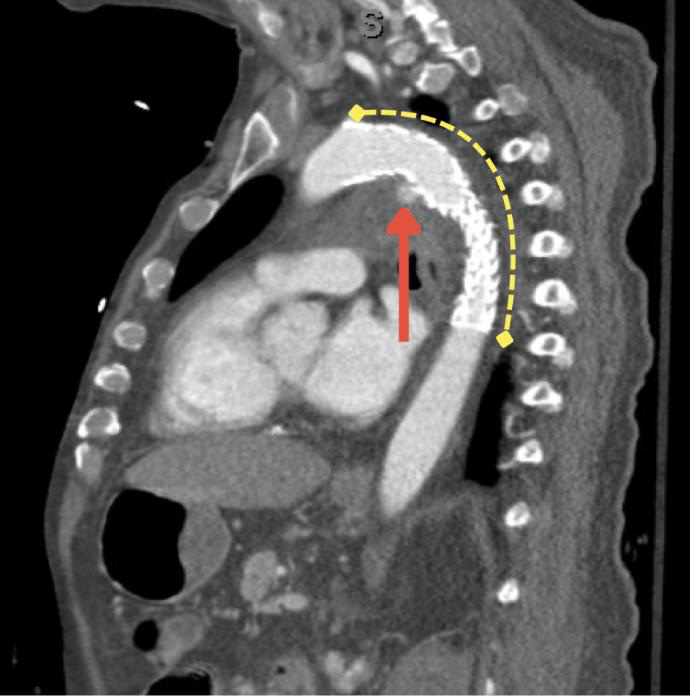
Computed tomography angiogram of the chest demonstrating the length of the endograft (broken yellow line) and endoleak arising from the anterior/inferior aspect of interval endograft repair of the aortic arch (red arrow).

A 70-year-old man with a past medical history of alcohol use disorder and thoracic aortic pseudoaneurysm presented to an outside hospital six days after discharge from a hospitalization for thoracic endovascular aortic repair, with hematemesis and hemorrhagic shock. Prior to arrival, the patient received 2 units of packed red blood cells, 2 units of fresh frozen plasma, 1 gram ceftriaxone, and 40 mg intravenous pantoprazole, and he was started on an octreotide infusion. On arrival at our ED his vital signs were as follows: blood pressure, 63/51 mm Hg with a mean arterial pressure of 55 mm Hg; heart rate, 69 beats per minute; respiration rate, 17 breaths per minute; and pulse oximetry, 98% on room air. He received an additional 1 unit of packed red blood cells, and his blood pressure improved to 90/78 mm Hg. Physical exam was notable for an awake and alert male, without distress, with abdominal tenderness to palpation in all four quadrants, and no active hematemesis. Computed tomography angiogram (CTA) revealed interval endograft repair of the aortic arch, endoleak present at the anterior/inferior aspect of the graft, and increased size of a superior mediastinal hematoma when compared to CTA seven days prior (Image 1).

After evaluation from surgical colleagues there was concern that the patient’s presentation might represent a variceal hemorrhage, especially given his alcohol use history. Given that endoleaks occur in 30% of patients after thoracic endovascular aortic repair, it was also considered that the CT findings might have represented anticipated sequela of his previous operation, rather than an aortoesophageal fistula.^8^ After arriving at the medical ICU, the patient underwent elective intubation for a planned EGD to evaluate for other etiologies of hematemesis. Shortly thereafter, but prior to EGD, the patient developed profuse pulsatile bleeding from his oropharynx accompanied by severe hypotension. Despite the initiation of a massive transfusion protocol, life-threatening hemorrhage continued. The patient’s blood pressure dropped to 50/45 mm Hg with only transient improvements with resuscitation. Within several minutes approximately 3 liters of blood were suctioned from the oropharynx.

*CPC-EM Capsule*

What do we already know about this clinical entity?

*Aortoesophageal fistula is an uncommon and rapidly fatal complication of thoracic endovascular aortic repair.*

What makes this presentation of disease reportable?

*This rare complication was successfully managed with the use of a Minnesota tube, highlighting that hemostasis can be achieved by nonspecialists at bedside.*

What is the major learning point?

*Clinicians should have a high index of suspicion in patients who present with a history of prior thoracic aortic or esophageal surgical interventions.*

How might this improve emergency medicine practice?

*Temporary hemostasis from an aortoesophageal fistula can be achieved with the use of a Minnesota tube or similar esophageal-gastric balloon tamponade device.*

**Image 2:**
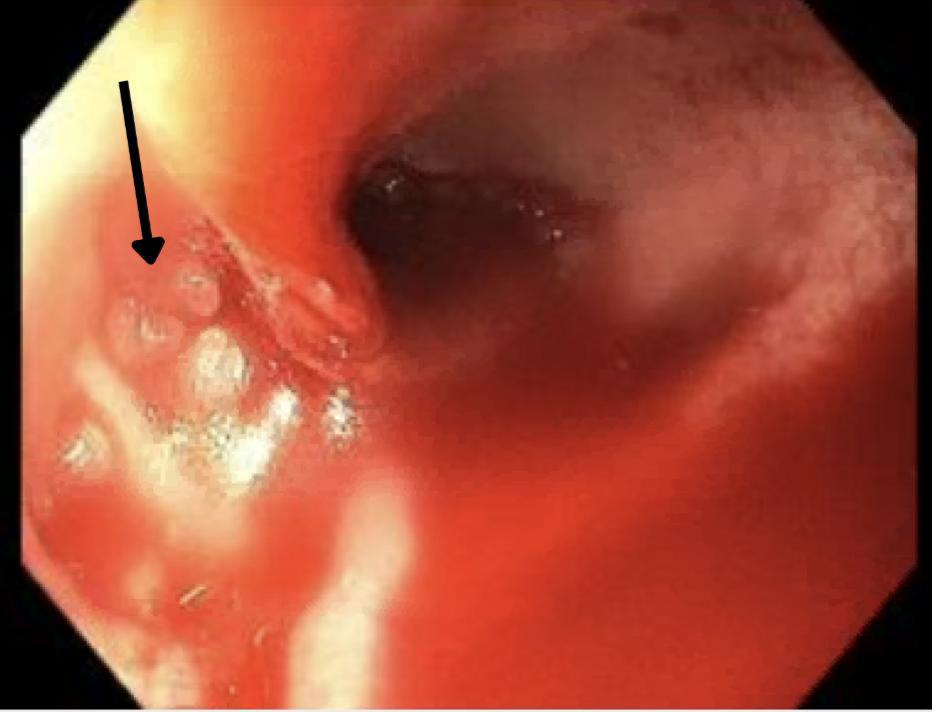
Profuse bright red blood and evidence of the outer metal frame (black arrow) of the aortic endograft in the esophagus seen during esophagogastroduodenoscopy.

The decision was made to place a Minnesota tube to achieve temporary hemostasis. A video laryngoscope was advanced into the vallecula until the posterior glottis was visualized. The Minnesota tube (bathed in ice) was advanced into the esophagus to 42 cm at the teeth. The gastric balloon was inflated, first with 200 mL of air and then pulled back until there was slight tension. The bleeding continued. The esophageal balloon was inflated gently until the bleeding in the oropharynx stopped, which occurred at 75 mL of air. Laryngoscopy revealed no active hemorrhage within the pharynx or upper esophagus. The gastric balloon was then totally deflated (no longer needed), and the esophageal balloon remained inflated. No ongoing blood was visualized when suction was applied to the gastric port. After the patient’s hemodynamics improved and temporary hemostasis had been achieved, EGD was performed. Endoscopy demonstrated active spurting from an arterial source, 23 cm from the incisors, with evidence of vascular mesh in the esophagus (Image 2). The Minnesota tube was then re-inserted, successfully bridging the patient to the operating room for endovascular repair of the fistula with placement of an additional thoracic aortic endograft.

## DISCUSSION

Aortoesophageal fistula is an uncommon but lethal cause of GI hemorrhage. In patients with prior thoracic aortic or esophageal intervention, hematemesis should always be considered a harbinger of aortoesophageal fistula until proven otherwise. While definitive management includes open or endovascular surgical intervention, use of balloon tamponade devices can be considered to achieve provisional hemostasis. As their use for aortoesophageal fistula is not a U.S. Food and Drug Administration-approved indication, careful consideration should be given to employing the device in the safest way possible. First, it is important to localize the source of hemorrhage to the esophagus and not the duodenum. Because most aortoenteric fistulas occur in the third or fourth segment of the duodenum, esophageal balloon tamponade is ineffective because the device cannot reach the site of hemorrhage.^9^ Further, these devices are not intended to be inflated in the small bowel. If the hemorrhage were suspected in the stomach, the gastric balloon could be inflated. However, there is a paucity of reports in the literature on the use of these devices for an aortogastric fistula, and best practice remains unclear. Primary aortogastric fistula represents only 2% of all aortoenteric fistulae.^10^ In our case, we had imaging suggesting a proximal area of hemorrhage and a graft terminating proximal to the gastroesophageal junction. This increased the probability of localizing the fistula to the esophagus.

Complications may arise with use of balloon tamponade devices in approximately 20% of patients because of balloon migration, overinflation, misplacement, or prolonged use. These include aspiration, ulceration, necrosis, and esophageal rupture.^11^ While the arterial pressure driving the hemorrhage in aortoesophageal fistula is significantly higher than the pressure of a varix in variceal hemorrhage, there is no evidence in the literature suggesting it is safe to exceed the balloon pressure and volume limits set by the manufacturers. Fortunately, most of these patients go to the operating room quickly once hemostasis has been achieved, minimizing total balloon inflation time.

Compared to previously published experiences, our case report illustrates that placement of a balloon tamponade device for aortoesophageal fistula can be performed successfully by nonspecialists. While management of our patient took place in the ICU, the merits of the case also extend to the ED. The critical nature of aortoesophageal fistulas and the associated high mortality demand a high index of suspicion and, at times, quick and life-saving intervention. Placement of balloon tamponade devices for variceal hemorrhage is already a skill within the scope of practice for intensivists and emergency physicians. These devices should also be considered an additional tool for management of severe non-variceal GI hemorrhage.

## CONCLUSION

Aortoesophageal fistula is a rare but deadly cause of gastrointestinal hemorrhage, typically leading to rapid exsanguination. In patients with prior thoracic aortic or esophageal intervention, aortoesophageal fistula should always be considered. Given the time-sensitive nature of the disease, temporary hemostasis can safely and adequately be achieved with a balloon tamponade device as a bridge to definitive treatment. Intensivists, emergency physicians, and other nonsubspecialists familiar with caring for the critically ill are well equipped to perform this procedure.
